# A Unified Framework to Prioritize RNA Virus Cross-Species Transmission Risk Across an Expansive Host Landscape

**DOI:** 10.3390/v18020211

**Published:** 2026-02-05

**Authors:** Di Zhao, Yi-Fei Wang, Zu-Fei Yin, Ya-Fei Wu, Hui-Jun Yu, Luo-Yuan Xia, Xiao-He Liu, Xiao-Ming Cui, Xiao-Yu Shi, Dai-Yun Zhu, Na Jia, Jia-Fu Jiang, Wu-Chun Cao, Wenqiang Shi

**Affiliations:** 1 Institute of EcoHealth, School of Public Health, Cheeloo College of Medicine, Shandong University, Jinan 250012, China; zellazdi@163.com (D.Z.);; 2 State Key Laboratory of Pathogen and Biosecurity, Academy of Military Medical Science, Beijing 100071, China; 3 School of Public Health, Nanjing Medical University, Nanjing 211166, China; 4 National Institute of Pathogen Biology, Chinese Academy of Medical Sciences & Peking Union Medical College, Beijing 100071, China

**Keywords:** RNA viruses, host prediction, genomic language model, virus–host interaction, cross-species transmission

## Abstract

RNA viruses exhibit high mutation rates and strong host adaptive capacity, posing major public health challenges. Although meta-transcriptomic studies have uncovered vast numbers of novel RNA viral sequences, identifying those with spillover risks remains difficult. Current virus host-prediction methods can only predict a narrow set of host labels at coarse taxonomic levels (e.g., kingdom or order), which hampers precise evaluation of cross-species transmission risk and may overlook potential zoonotic hosts. To overcome these limitations, we developed UniVH, a unified virus–host association prediction framework trained on an exceptionally broad spectrum of 90 viral families and 240 host families, enabling robust prediction even for phylogenetically distant or data-scarce hosts. UniVH achieved a host prediction accuracy of 71.2% for novel viruses discovered after 2020, representing a 15.3% improvement over conventional BLASTp-based homology approaches. Feature interpretation revealed that viral structural genes and host immune- and metabolism-related genes contributed most significantly to predictive performance. Model predictions indicated widespread host-range expansion, with 20 mammalian virus families doubling their documented mammalian host ranges and several showing marked increases in viruses with human-infection potential. This unified, interpretable framework represents an important methodological advance for future RNA virus spillover-risk evaluation and emerging virus prioritization.

## 1. Introduction

RNA viruses evolve rapidly and possess remarkable host adaptive flexibility, making them major threats to global public health. They include high-impact pathogens such as SARS-CoV-2, influenza viruses, and Ebola virus, all of which have caused substantial harm to human society. A large proportion of these emerging infectious pathogens in humans are zoonotic, with many viruses originating from wild animals [1]. With the widespread adoption of meta-transcriptomic sequencing, vast numbers of novel viral sequences have been identified from wildlife [2,3], smuggled animals [4], arthropods [5], and environmental samples [6]. These newly discovered viruses typically share limited sequence similarity with known references, and we typically do not know their ecological hosts, transmission routes, and pathogenic potential. Determining which of them represent high-risk viruses has therefore become a critical challenge for preventing the next pandemic.

Accurate prediction of the host range for novel viruses remains highly challenging. Host range is inadequately characterized even for the best-studied viruses. Traditional wet-lab approaches require obtaining isolates or constructing pseudoviruses, which are low-throughput, time-consuming, and unsuitable for large-scale analysis. Current computational methods predominantly rely on viral genomic composition features (e.g., k-mers, codon usage bias) to predict coarse host categories, resulting in limited resolution that typically does not exceed the order level [7]. In addition, most existing models are trained on viruses with abundant available sequences or on a narrow set of host taxa [8,9], which restricts their generalizability to novel emerging viruses or less studied host species.

The establishment of viral infection and replication within a host is orchestrated through a coordinated series of protein–protein interactions between viral and host factors. Viruses exploit these interactions to hijack host cellular pathways—facilitating cell entry and exit, viral genome replication, intracellular trafficking, and evasion of immune defenses [10]. To achieve such host–pathogen interactions, many viruses employ molecular mimicry, imitating host proteins at the sequence, motif, or structural levels [10,11]. Consequently, host characteristics—particularly genomic and proteomic features—are critical for predicting viral host ranges. However, the inherent complexity of host genomes and limited availability of host genomes remain major bottlenecks that constrain predictive accuracy. With the development of genomic and protein language models [12,13], which can effectively capture high-level features of protein sequences and structures, it has become possible to perform unified modeling and analysis of viral and host genes.

In this study, we developed a host-adaptation prediction framework for novel viruses in the context of emerging infectious disease surveillance. We modeled RNA virus–host associations as a unified binary classification problem by matching viral and host genomic features across diverse RNA virus families, enhancing generalization to newly discovered viruses. Our method leveraged genomic language models to embed viral and host genes into a shared feature space and employed function-guided attention mechanisms to efficiently aggregate whole-genome signals. By further integrating ecological and geographic metadata, the framework enabled multimodal inference. This approach provided strong generalization and broad-spectrum applicability, supporting the prediction of cross-species transmission risks for highly variable or phylogenetically distant novel viruses.

## 2. Materials and Methods

### 2.1. Integrated Construction of Virus–Host Association Datasets

To comprehensively assemble the virus–host association dataset, we first retrieved viral metadata from the NCBI Virus database (https://www.ncbi.nlm.nih.gov/labs/virus/vssi/#/, accessed on 27 May 2025), which provides host information and sampling locations for each viral nucleotide record in NCBI GenBank. We further incorporated data from the Virus–Host Database (VHDB) [14] (https://www.genome.jp/virushostdb/, accessed on 30 May 2025), which aggregates manually curated virus–host species-level associations. To resolve taxonomic inconsistencies between viruses and hosts, we standardized all classification information using the NCBI Taxonomy database [15] (as of 9 June 2025) and the Taxonkit tool (v0.8.0), ensuring consistent species-level taxonomy and effectively reducing data redundancy while improving quality. Furthermore, host–virus mappings were validated using the host.dmp file from the NCBI Taxonomy database (Taxdump), which systematically records correspondence between viruses and their host categories.

Our classification model (see next section) requires viral and host genomic sequence information for each virus–host pair. We batch-downloaded all complete nucleotide sequences (“Complete Nucleotides”, as of 27 May 2025) of RNA viruses from the NCBI Virus database, yielding 3,262,043 sequences. For segmented RNA viruses, we fully considered the complexity of their genome structures. Related nucleotide records of the same virus species were grouped based on “Assembly”, “Isolate”, and “Strain” metadata fields, and segments from the same group were consolidated into a single record to ensure all essential segments were included. To rigorously ensure the quality of viral sequences, we used CheckV (v1.0.3) [16]. CheckV evaluates viral contig quality by integrating protein-coding gene prediction and homology searches against reference viruses. It estimates genome completeness using genome-length information and terminal-signature evidence. In addition, it identifies putative host-derived (cellular) regions to assess contamination and to delineate proviral boundaries. Only sequences labeled “High-quality” or “Complete” in the checkv_quality output were retained, thereby minimizing the inclusion of fragmented or host-contaminated viral sequences in downstream analyses. Further, considering the potential sequence variability among isolates and samples of the same virus species, we performed species-level clustering. Specifically, we used CD-HIT (v4.7) [17] with a 99% sequence identity threshold to cluster viral sequences at the species level, retaining a representative sequence for each cluster and removing redundancy. For the host in each virus–host pair, we queried the NCBI Assembly database (as of 30 May 2025) and downloaded the corresponding host genome coding sequence (CDS) files. Ultimately, 24,354 high-quality viral sequences and 525 host genome sequences were included, providing a standardized and traceable dataset for subsequent virus–host interaction studies.

We generated a high-confidence negative dataset by excluding all known virus–host associations recorded in the NCBI Virus database and VHDB. During negative sample construction, we ensured that the distributions of viruses and hosts in the negative set matched those in the positive set. For each virus, host species belonging to the same family as their known hosts were excluded to avoid false negatives, as phylogenetically close hosts are more likely to be infectable. Host candidates were then selected according to a prioritized taxonomic hierarchy—same order, same class, same phylum, and same kingdom. To mitigate the imbalance caused by cluster frequency in the positive dataset, we performed up-sampling of low-frequency clusters (*n* < 10) in the train dataset. Following this strategy, we constructed a negative dataset at a 1:1 ratio relative to the positive dataset.

### 2.2. Geographical Environmental Data

For the host and virus in each pair, host geographical distribution was obtained from the Global Biodiversity Information Facility (GBIF) through GBIF Occurrence Downloads (including https://doi.org/10.15468/dl.cv6a9j, https://doi.org/10.15468/dl.ykmpv7, and https://doi.org/10.15468/dl.9j6c2w; accessed on August 2025), while viral geographical distribution was extracted from the “Geo_location” field of the virus records in the NCBI Virus database. Both virus and host geographical coordinates were then mapped to the world’s major biogeographic realms [18], which include the Nearctic, Palearctic, Neotropical, Afrotropical, Indomalayan, Australasian, Oceanian, and Antarctic biogeographic realms. To avoid misclassification caused by marginal or occasional sightings, we considered only the primary realms that together accounted for the top 90% of occurrence records for each host species. Virus and host distributions were further projected onto a 50 km grid. For each virus–host pair, spatial overlap between the virus and host was quantified in two ways: ${\mathrm{overlap}}_{\;V}=\frac{{\mathrm{grid}}_{V,H}}{{\mathrm{grid}}_{V}}$, ${\mathrm{overlap}}_{H}=\frac{{\mathrm{grid}}_{V,H}}{{\mathrm{grid}}_{H}}$. Here, grid*_V_*_,*H*_ represents the number of grid cells where both the virus and the host are present, while grid*_V_* and grid*_H_* represent the number of grid cells occupied by the virus and the host, respectively. In addition, we calculated the average geographical distance between the virus and host species based on the latitude and longitude of the centers of their occupied grid cells.

### 2.3. Unified Virus–Host Prediction Model

We developed a unified binary classification framework to predict virus–host associations across diverse RNA virus families and host taxa. The model integrates complete sets of viral and host coding DNA sequences (CDS) together with ecological and geographical metadata. Host CDS were annotated into 8377 gene families and assigned to five top-tier KEGG functional categories [19], while viral CDS were grouped into 706 families and categorized into five VOGDB VFAM classes [20]. Each viral CDS and the representative CDS of each host gene family (the longest CDS) were encoded as 2560-dimensional embedding vectors using LucaOne [13]. For each virus–host pair, the model learned a trainable weight for every viral or host gene family, reflecting its relative contribution to infection prediction. Weighted family embeddings within each functional category were aggregated—normalized by softmax—to obtain a category-level representation. The concatenated category representations formed a tensor that was passed into a transformer encoder layer (nn.TransformerEncoderLayer in PyTorch 1.12.1 [21]) comprising four-head self-attention, a feed-forward network, dropout (rate 0.1), and ReLU activation. This architecture enables the model to capture complex higher-order interactions between viral and host functional categories. The output of the transformer encoder was flattened and concatenated with one-hot-encoded viral and host metadata. These combined features were then passed through a two-layer fully connected classification head to produce the final prediction of virus–host associations.

The model was trained using binary cross-entropy loss (BCEWithLogitsLoss) and optimized with Adam. Training was performed with a batch size of 128 and GPU-accelerated collate functions for efficient data loading. Model performance was evaluated on the validation set at the end of each epoch using accuracy, F1 score, precision, recall, PR-AUC, and ROC-AUC, with results visualized through PR and ROC curves.

### 2.4. Comparative Models Based on Genomic Composition and Sequence Similarity

For model comparison, we built classification models based on genome composition features derived from viral and host coding sequences, including nucleotide bias, dinucleotide bias, and codon-pair bias. Specifically, we counted the five nucleotides (A, C, G, T, N) and calculated their frequencies in the coding region as nucleotide bias features. For the 16 possible dinucleotides, we computed their relative preferences and separately tallied dinucleotide preferences for “bridge” (bridging codon boundaries) and “non-bridge” positions to capture structural distributions. Codon-pair bias was evaluated using codon pair scores (CPS) for all 4096 possible codon-pair combinations (64 × 64), with stop codons included to ensure completeness [7]. For unobserved codon or amino acid pairs, we imputed the average score across isolates or flagged them as missing to avoid null value impacts on the analysis. Additionally, codon usage preferences for all 64 codons and 21 amino acids (including stop codons) were calculated. In total, we compiled 48 dinucleotide bias features, 4096 codon pair scores, 64 codon usage features, and 21 amino acid preference features, amounting to 4229 genome composition features. Using these features, we trained GBM and Transformer models. The GBM model was implemented using LightGBM [22], optimized through gradient boosting decision trees with a range of key hyperparameters (such as learning_rate, max_depth, etc.), and evaluated systematically using early stopping and multiple metrics (such as accuracy, AUC, F1, etc.) to assess model performance. The Transformer model was implemented using PyTorch and consists of an input fully connected layer, two layers of Transformer encoders (128-dimensional, 8-head attention, 256-dimensional feed-forward networks), a classification layer, and a sigmoid output, enabling high-dimensional modeling of 4229 genomic composition features. During training, the Adam optimizer and early stopping mechanism were used. Both models were trained and evaluated according to a unified data splitting strategy to ensure comparability and fairness of the results.

We further introduced BLASTp (v2.7.1+) [23] for model comparison. The key assumption underlying this approach is that sequence similarity implies similar infectivity among viruses. Predicted protein sequences from training viruses were concatenated to construct a BLAST protein database, and predicted proteins from test viruses were used as queries. BLASTp hits were retained if they met E-value < 1 × 10^−5^ and query coverage > 50%. To aggregate protein-level alignments to the virus level, for each (query virus, training virus) pair, we kept the single best protein alignment (highest bit score) across all protein–protein matches between their proteomes. The host range of a query virus was then inferred by transferring/aggregating host annotations from the matched training viruses. For evaluation, we report BLASTp results under two aggregation settings—(i) retaining all qualified training-virus matches (all-cover) and (ii) retaining only the top-ranked matches—both of which serve as baselines for comparison with our model.

## 3. Results

### 3.1. Integrated Genomic and Geographic Data for Each RNA Virus–Host Association

**Figure 1:**
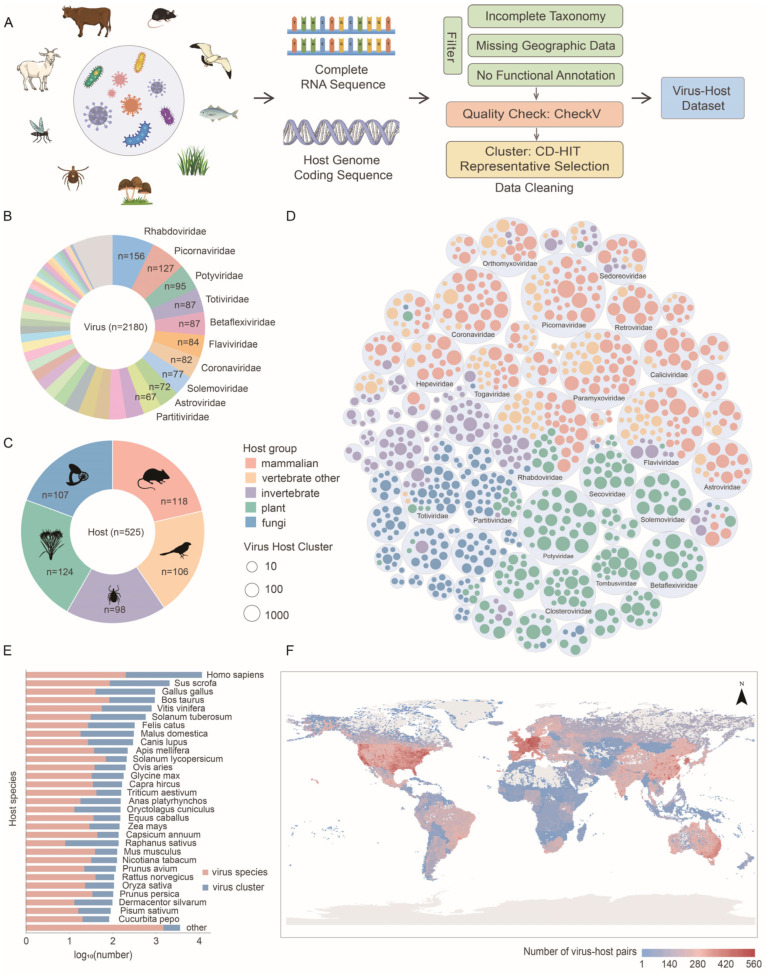
RNA virus–host interaction landscape across host taxa and geographic regions. (**A**) Data acquisition, curation, and quality control of the RNA virus–host association data. (**B**) Composition of viral species grouped at the family level. Each sector corresponds to a viral family, with *n* indicating the number of virus species. (**C**) Composition of host species grouped by major host categories. Each sector represents a host group, with *n* indicating the number of host species. Silhouette icons denote representative taxa for each group. (**D**) Diversity and interaction patterns between virus families (large light gray circles) and host families (small colored circles). Viral sequences were clustered at 99% sequence identity. The size of each small circle reflects the number of unique virus-cluster–host associations within each host family. Circles are colored by host category (fungi, plants, invertebrates, other vertebrates, and mammalian vertebrates). The top 20 virus families with the highest association counts are labeled. (**E**) Bar chart of virus diversity in major host species. Pink bars indicate the number of viral species associated with each host species, while blue bars represent the number of viral clusters. Host species are ranked by their total number of virus associations, with the top 30 species shown. (**F**) Global heat map of the virus-cluster–host association occurrence. The color indicates the number of virus-cluster–host associations mapped to the given 50 km grid.

To systematically compile association information between RNA viruses and hosts, we first constructed paired RNA virus–host association data based on the NCBI Virus database [24]. Since viral host records in NCBI do not necessarily indicate infection, we use the term “association” to refer to this relationship. Then, we downloaded complete nucleotide sequences for all RNA viruses in the paired data and integrated corresponding host genome data from the NCBI Assembly database. After data processing, we obtained a total of 3071 unique virus–host species pairs (Figure 1A). The dataset covered 2180 viral species spanning 90 confirmed viral families (Figure 1B), representing 73.17% of all RNA families, with an additional 180 viral species not yet assigned to any established viral family. In addition, the dataset included 525 host species encompassing 240 host families, including vertebrates, invertebrates, plants, and fungi (Figure 1C).

Given that RNA viruses generally exhibit high mutation rates and genomic diversity, sequences within the same viral species may show considerable sequence variation. For example, the *Orthoflavivirus nilense* contained 976 complete sequences with nucleotide similarity as low as 76.65%; the *Orthoflavivirus denguei* contained 1500 complete sequences with nucleotide similarity as low as 75.51%. Therefore, we performed sequence clustering analysis at the viral species level using a 99% sequence similarity threshold. This effectively reduced sequence redundancy and obtained representative viral clusters. After the above processing steps, we generated 24,354 virus-cluster–host pairs. The Rhabdoviridae family exhibited the broadest host range, being associated with 65 host families spanning five kingdoms (Figure 1D). In addition, Retroviridae viruses associated with Hominidae showed the greatest interaction diversity, comprising 3894 clusters. Overall, the dataset was highly imbalanced across the 525 host species, with the top 20 host species accounting for 80.71% of all associations (Figure 1E). This pronounced skew underscores the need for a model with strong generalization capability to accurately predict the association of novel viruses and underrepresented hosts.

To characterize geographic patterns, we projected each virus–host association onto overlapping 50 km spatial grid cells using NCBI accession metadata for virus locations and host occurrence records derived from the Global Biodiversity Information Facility (GBIF, https://www.gbif.org/) database. Virus–host associations showed highly uneven global distributions. The highest densities occurred in North America, Western Europe, and East Asia—regions with extensive virus detection and sequencing initiatives. In contrast, vast areas of Africa, South America, and Southeast Asia displayed markedly fewer documented associations despite their high wildlife diversity, revealing major gaps in pathogen surveillance and host sampling coverage (Figure 1F).

### 3.2. Unified Prediction Framework Across Diverse RNA Viruses and Hosts

**Figure 2:**
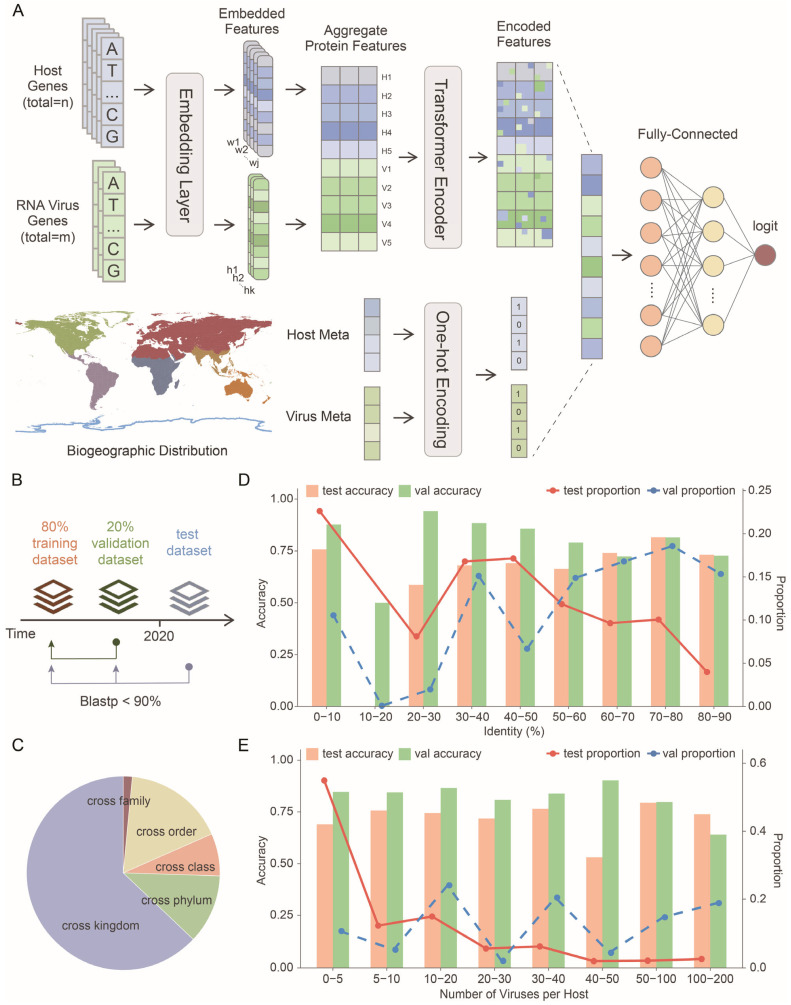
Model architecture, training, and evaluation of UniVH. (**A**) Schematic overview of the model framework. Virus and host genes were embedded into a unified representation space using a genomic language model. Gene embeddings were aggregated according to gene functional category, weighted, and dimensionally reduced, and then concatenated across all categories. The fused features were input into a Transformer encoder, where multi-head self-attention captured relationships among function categories. The encoder output was concatenated with ecological and geographic information of the virus and host, followed by two fully connected classification layers to predict binary virus–host associations. (**B**) Partition of the train, validation, and test datasets according to submission date and sequence similarity. (**C**) The distribution of negative samples compared to positive datasets. (**D**) Model performance across different virus sequence similarity groups on validation and test datasets. Bars show accuracy (left y-axis) on the test set (orange) and validation set (green) for each sequence identity interval, while lines indicate the proportion of samples within each interval (right y-axis) in the test (red) and validation (blue) datasets. (**E**) Model performance across hosts stratified by the number of associated viruses in the validation and test datasets. Bars show accuracy (left y-axis) on the test set (orange) and validation set (green) for each sequence identity interval, while lines indicate the proportion of hosts within each interval (right y-axis) in the test (red) and validation (blue) datasets.

Based on the RNA virus–host association data described above, we developed a unified binary classification model for predicting virus–host associations (UniVH) across diverse RNA virus families and host taxa using the complete viral and host gene repertoires together with their geographic and ecological metadata (Figure 2A). We first employed the genomic language model LucaOne [13] to encode 1.72 million host genes and 93,332 viral genes into high-dimensional embeddings that capture their contextual and structural properties. Host genes were grouped into 8377 gene families and further assigned to five major KEGG functional categories [19]: Metabolism (21.91%), Genetic Information Processing (19.54%), Environmental Information Processing (19.63%), Cellular Processes (16.36%), and Organismal Systems (22.56%). Viral genes were grouped into 706 gene families and categorized into five VOGDB VFAM-based functional classes [20]: virus structure genes, virus replication genes, multifunctional genes, host/virus-beneficial genes, and genes of unknown function, accounting for 41.13%, 11.33%, 30.36%, 7.37%, and 9.82% of all viral genes, respectively. For each virus–host pair, the model learned a trainable weight for each viral or host gene family, reflecting its relative contribution to prediction. Embeddings within the same functional class were subsequently aggregated to obtain class-level representations. The five host and five viral category embeddings were then aligned and fed into a transformer module [25] followed by a classification head to capture high-order cross-class interactions. To further enhance discriminative power, additional metadata—such as geographic overlap ratio, ecological realm, and other host-virus attributes—were encoded as one-hot or scalar vectors and jointly integrated into the classification head along with embedding features, thereby improving model robustness and generalization performance.

The entire dataset was strictly partitioned into training, validation, and test sets based on both sequence similarity and record submission time to prevent data leakage. Viral species discovered after 2020 were assigned to the test set, whereas species identified before 2020 were split into training and validation sets at an 8:2 ratio at the species level. To further ensure generalization, viral protein sequence similarity between the training set and either the validation or test sets was constrained to be below 90% (Figure 2B). For negative dataset construction, we randomly paired viruses with unknown hosts according to a prioritized taxonomic hierarchy—same order, same class, same phylum, and same kingdom, facilitating the model’s ability to distinguish virus–host associations at varying evolutionary distances (Figure 2C). As a result, the final training set comprised 52,256 unique virus-cluster–host records, while the validation and test sets contained 2633 and 1680 records, respectively.

On the test set, the model achieved an accuracy of 71.19%, an ROC-AUC of 0.7573, a PR-AUC of 0.7140, an F1 score of 0.7423, a precision of 0.6833, and a recall of 0.8124, demonstrating strong discriminatory power for virus–host range prediction. Stratified evaluation across major host categories showed accuracies of 83.16% for vertebrate mammals, 73.53% for other vertebrates, 63.53% for invertebrates, 76.32% for plants, and 68.67% for fungi. These results indicate that the model effectively distinguishes host types across multiple biological kingdoms and exhibits robust generalization capability.

UniVH generalized well across novel viruses and rare hosts. Even when evaluated on viruses sharing less than 10% protein sequence identity with those in the training set, UniVH maintained an accuracy of 75.79%. These highly divergent viruses comprised a substantial proportion (22.62%) of the test set (Figure 2D). UniVH demonstrated robust accuracy for both common hosts (with >50 known associated viruses) and rare hosts (with <10 known associated viruses), which accounted for 90.29% of all host species (Figure 2E). This robust performance indicates strong generalization to novel viruses and underrepresented host taxa, supporting improved assessment of zoonotic risk.

### 3.3. Enhanced Predictive Accuracy Across Diverse Viral Families

To further evaluate the performance of UniVH, we compared it with traditional approaches based on genomic composition features and BLASTp similarity search [23]. Specifically, we quantified 4229 genome-composition features for both viruses and hosts—including all possible codon pairs, dinucleotides, and codon and amino acid usage biases [7]—and trained models using gradient boosting machines (GBM) [26] and transformer-based architectures. In parallel, we applied BLASTp to search each test viral gene against the training set to infer host range based on sequence similarity.

**Figure 3:**
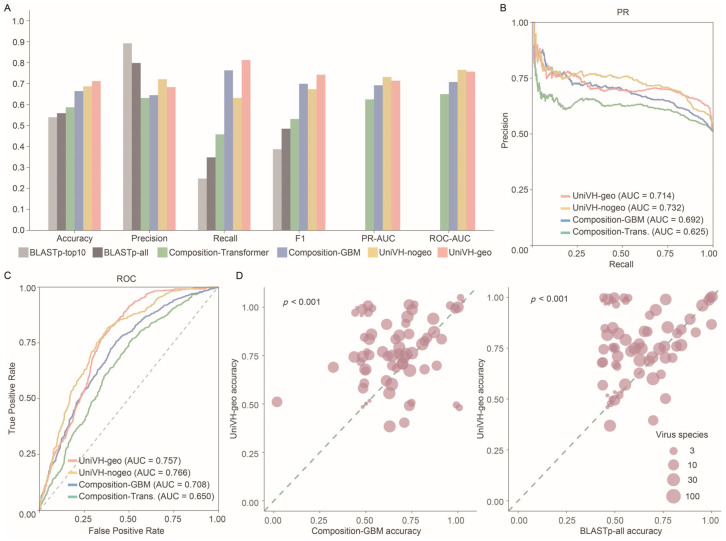
Benchmarking of UniVH against other virus host prediction methods. (**A**) Accuracy, precision, recall, F1 score, PR-AUC, and ROC-AUC for UniVH and other models. Composition-GBM: Genome composition features trained with gradient boosting models. Composition-Transformer: Genome composition features trained with Transformer models. UniVH-nogeo: UniVH trained without geographic features. UniVH-geo: UniVH trained with geographic features. BLASTp-top10: Host inference using the top 10 BLASTp hits. BLASTp-all: Host inference using all BLASTp hits. (**B**) PR curves for genome composition-based models and UniVH. (**C**) ROC curves for genome composition-based models and UniVH. Dashed lines indicate the performance expected by random chance. (**D**) Performance comparison of UniVH across different virus families against genome composition-based models and BLASTp. Dashed lines indicate equal accuracy between the two methods.

The results (Figure 3A–C) showed that UniVH markedly outperformed the composition-GBM, composition-transformer, and BLASTp approaches across accuracy, F1 score, PR-AUC, and ROC-AUC. UniVH without geographic features achieved the highest PR-AUC (0.7316), followed by UniVH with geographic features (0.7140), composition-GBM (0.6920), and composition-transformer (0.6248), whereas BLASTp exhibited the lowest accuracy among all methods. Across the 69 viral families evaluated, UniVH achieved higher accuracy than the composition-GBM model in 43 families and comparable accuracy in 14 families (Figure 3D). UniVH further outperformed BLASTp in 39 families. These results demonstrate that a deep-learning framework combining gene-level contextual embeddings with functional aggregation captures virus–host relationships more effectively than sequence-composition or similarity-based approaches, yielding superior discriminative power and generalization for host-range prediction.

### 3.4. Key Gene Families Driving RNA Virus–Host Prediction

**Figure 4:**
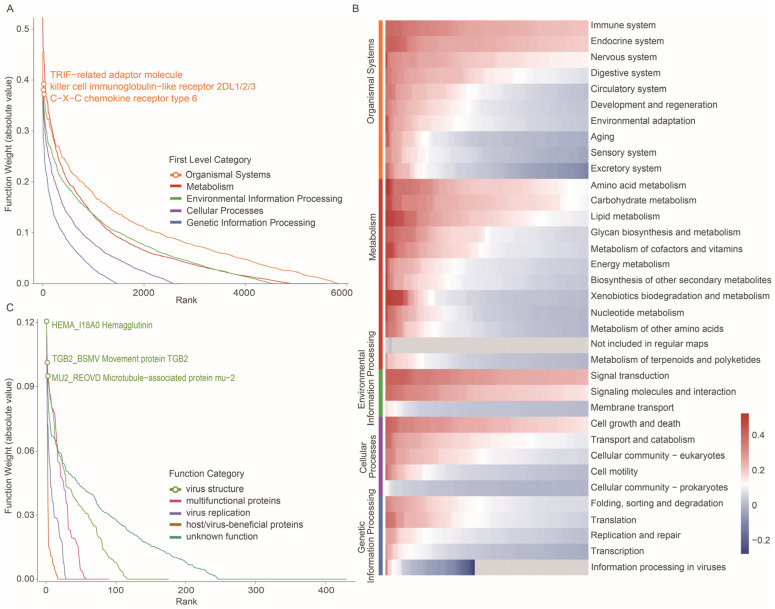
Key gene families driving RNA virus-host prediction. (**A**) Distribution of learned functional weights for host gene families grouped by KEGG first-level functional categories. Gene families are ranked along the x-axis by absolute weight, and the y-axis shows the corresponding functional weight (absolute value). (**B**) Heatmap of learned weights for host gene families across KEGG second-level functional categories in UniVH. The top 100 gene families within each second-level category are shown. The color bar on the left denotes the KEGG first-level category, with category names listed on the right. (**C**) Distribution of learned functional weights for viral gene families grouped by VOGDB functional categories. The x-axis indicates the gene family rank, and the y-axis represents the assigned functional weight (absolute value).

In our UniVH framework, the functional weights of gene families provided crucial evidence for analyzing the model’s discriminative mechanisms and its underlying biological significance. We systematically surveyed the learned weights of host and virus gene families to identify which functional categories and gene families play central roles in the virus–host discrimination task. The weights allocated by UniVH exhibited a distinct long-tail distribution for both host and virus functional categories, indicating that the discrimination process heavily depends on a small subset of high-weighted functions. Notably, on the host side, the weights for the genes in the “Organismal Systems” and “Metabolism” categories were markedly higher than those of other first-level categories. At the secondary classification level, genes involved in the immune system, amino acid metabolism, and signal transduction stood out (Figure 4A,B). For example, in the “Toll-like receptor signaling [PATH:ko04620]” pathway, some gene weights reach as high as 0.39, while in the “Natural killer cell mediated cytotoxicity [PATH:ko04650]” pathway, certain genes show weights up to 0.38. Specifically, high-weight genes included the TRIF-related adaptor molecule (K05409) and Killer cell immunoglobulin-like receptor 2DL1/2/3 (K07981), which belong to these immune pathways and play central roles in antimicrobial defense, inflammatory regulation, and platelet-mediated immune responses [27,28].

For viruses, we further analyzed the functional weight distribution of various viral families (VFAM) in the VOGDB database (Figure 4C). The results show that these families also exhibit a long-tail distribution. Among them, unknown function and virus structure categories included the greatest numbers of high-weight families. For example, within the virus structure category, the hemagglutinin (sp|Q9WFX3|HEMA_I18A0) can reach a weight of 0.12, and the movement protein TGB2 (sp|P04869|TGB2_BSMV) can reach 0.10. Hemagglutinin is an important glycoprotein located on the viral envelope surface, mediating the binding of viruses to host cell receptors and facilitating viral entry, playing a critical role in infection and cross-species transmission [29]. The movement protein TGB2 is a structural component of plant viruses, responsible for the cell-to-cell movement and systemic infection of viruses within plant tissues, providing the foundation for viral spread [30]. The significant contribution of these high-weight families illustrates that the model strongly relies on recognition and invasion mechanisms mediated by structural proteins in predicting virus–host relationships.

### 3.5. Host-Range Expansion and Key Reservoirs Underlying Zoonotic Risk

**Figure 5:**
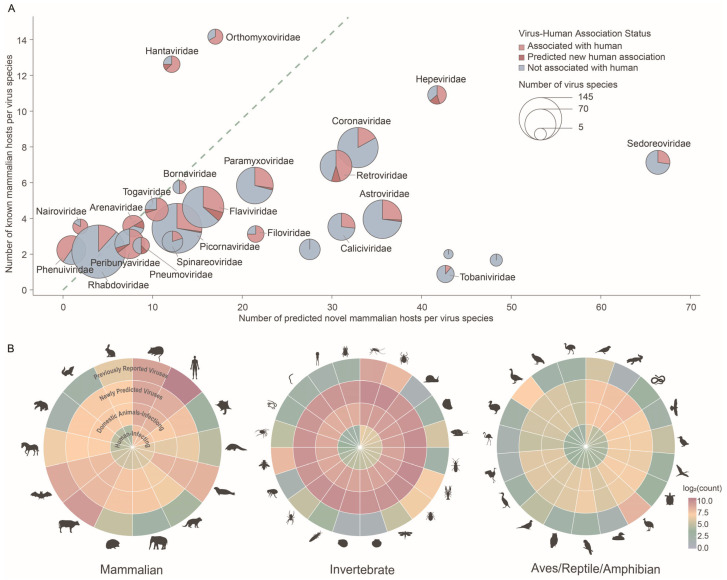
Model-predicted virus–host association patterns from viral and host perspectives. (**A**) Numbers of known and newly predicted human-associated viruses within each virus family. Circle size indicates the total number of virus species in each family, subdivided to show species already associated with humans, newly predicted to be associated with humans, and species not associated with humans. The dashed line indicates that the number of newly predicted human-associated viruses is twice the number of known human-associated viruses in each family. (**B**) Distribution of viruses across selected major host order groups. Each panel is represented by concentric circles (outer to inner) showing: (1) the number of viruses previously reported to be associated with the host group; (2) the number of newly predicted viruses associated with the host group; (3) the number of viruses predicted to be associated with economically important domestic animals; and (4) the number of viruses predicted to be associated with humans. Silhouettes represent the corresponding host order groups.

To assess the potential for viral host-range expansion in mammals, we compared documented hosts with model-predicted hosts across 25 mammalian virus families. The analysis revealed substantial expansion potential in most virus families: 20 out of 25 exhibited predicted new host ranges that doubled their documented ranges (Figure 5A). Among these, Astroviridae, Caliciviridae, and Sedoreoviridae exhibited 9.00-, 8.79-, and 9.30-fold increases, respectively, in the number of predicted novel mammalian hosts. In addition, Flaviviridae, Retroviridae, and Peribunyaviridae showed newly predicted human-infective viruses, with six, four, and two viruses, respectively.

From the host perspective, the total number of associated virus species increased by approximately 2.38-fold in mammals and 16.72-fold in non-mammalian vertebrates, followed by 42.75-fold in invertebrates, 49.45-fold in fungi, and 15.10-fold in plants. According to model predictions, several host groups harbored disproportionately large numbers of viruses with human-infection potential (Figure 5B). In mammals, Primates and Rodentia were the major reservoirs of potential human-infective viruses. In invertebrates, Diptera and Ixodida carried substantially more potential human-infective viruses than other arthropod groups, consistent with their roles as important vectors in zoonotic transmission [5,31]. These findings indicate that taxonomic groups such as Rodentia, Diptera, and Ixodida not only harbor high viral diversity but also act as key high-risk reservoirs and vectors, underscoring their central role in viral spillover and the prevention of emerging zoonotic diseases.

## 4. Discussion

RNA viruses evolve rapidly owing to inherently high mutation rates and error-prone replication, enabling rapid adaptation and frequent cross-species transmission that drive many emerging infectious diseases. Accurately predicting their potential host range, however, remains a central challenge. In this study, we present a unified modeling framework applicable across diverse RNA virus families, enabling fine-scale host discrimination. Our results showed that representing virus and host genes through embeddings, combined with aggregated functional classifications, allowed effective extraction of informative features from large, heterogeneous host genomes. This strategy markedly improved predictive accuracy and generalization, yielding robust performance across viral families and broad host taxa. Moreover, model-derived key features revealed mechanistic links between viral structural genes and host immune- and metabolism-related genes, highlighting molecular determinants of host specificity. Together, these findings provided a mechanistic basis for understanding host specificity in RNA viruses and established a generalizable computational framework for anticipating host adaptability in newly emerging viruses.

The RNA virus–host association dataset constructed in this study primarily relied on virus records from the NCBI Virus database. Specifically, the virus–host links documented in NCBI do not distinguish among infection, carriage, symbiosis, or other interaction types. It is important to emphasize that our use of the term “association” reflects a broad description of virus–host relationships rather than a strict biological interaction. Importantly, our framework serves as a scalable computational pre-screening tool that helps prioritize high-risk virus–host pairs for downstream laboratory validation. By narrowing the candidate space, the model can substantially reduce experimental workload and guide more targeted virological, ecological, and epidemiological investigations.

Accurate host prediction for novel RNA viruses remains highly challenging for several reasons. First, RNA viruses are extremely diverse, yet we still lack a systematic understanding of their infection mechanisms. For instance, cellular receptors for the majority of RNA viruses remain unknown [32], and this mechanistic knowledge gap limits our ability to model virus–host interactions in a biologically faithful manner. Second, for newly discovered viruses, both sequence data and experimentally validated host labels are extremely limited. Existing models—particularly genome composition-based approaches—perform well on a small set of extensively characterized viruses such as influenza and SARS-CoV-2 [33,34], where major host categories (e.g., human, avian, or swine) can be predicted reliably thanks to rich historical data. However, for entirely novel viruses, the current lack of known hosts and training labels prevents high-accuracy modeling. Third, while positive infection records are systematically archived, non-infection (negative) virus–host pairs are rarely documented, resulting in a critical scarcity of labeled negative samples for supervised learning.

The performance of the proposed model is expected to further improve as sequencing resources continue to grow. First, the rapid accumulation of meta-transcriptomic datasets is steadily expanding the catalog of known virus–host associations. Second, although our current model includes only 525 host species, ongoing large-scale genome initiatives—such as the Bird 10,000 Genomes Project [35], the i5K initiative (Sequencing 5000 Arthropod Genomes) [36], and Bat1K [37]—will substantially enrich available host genomic information and enhance model robustness. Third, previous studies have demonstrated that integrating geographic information can markedly improve pathogen-host association predictions [8]. In our model, the incorporation of geographic information improved predictive accuracy to 71.19%, underscoring its value. Currently, viral geographic annotations are often coarse—frequently limited to country-level descriptors—and distribution records for many host species in GBIF remain incomplete. As these datasets become more comprehensive and better resolved, the performance and taxonomic precision of UniVH are expected to improve accordingly.

Our model has also captured some key features underlying pathogen–host interactions. Classical theories suggest that codon usage and GC content similarity between viruses and hosts drive adaptation [38]. We found that codon usage-based prediction achieved only 66.43% accuracy, whereas our embedding-based model reached 71.19%, indicating the acquisition of higher-level, more informative features. We speculate the model may, for example, be learning aspects akin to “viruses mimicking host short linear motifs” [11], which merit further investigation. It should be noted, however, that the model cannot currently resolve fine-grained protein–protein interactions, which may be addressed in future work leveraging tools such as AlphaFold [39].

In summary, we introduce a unified virus–host modeling framework that leverages full viral and host gene repertoires to achieve fine-grained and broadly generalizable host-range predictions for RNA viruses. This approach establishes a scalable foundation for the early detection of high-risk emerging viruses and strengthens future pathogen surveillance efforts.

## Data Availability

The datasets used for model training and prediction are available at Zenodo (https://doi.org/10.5281/zenodo.17505409, accessed on 2 February 2026). This Zenodo repository also provides viral and host gene data, viral and host metadata, and the benchmark datasets used in this study. The relevant codes are available at GitHub (https://github.com/Z-099/UniVH-model, accessed on 2 February 2026).
